# Protective Effect of Shikimic Acid against Cisplatin-Induced Renal Injury: In Vitro and In Vivo Studies

**DOI:** 10.3390/plants9121681

**Published:** 2020-12-01

**Authors:** Jinkyung Lee, Quynh Nhu Nguyen, Jun Yeon Park, Sullim Lee, Gwi Seo Hwang, Noriko Yamabe, Sungyoul Choi, Ki Sung Kang

**Affiliations:** 1 College of Korean Medicine, Gachon University, Seongnam 13120, Korea; jklee9441@hanmail.net (J.L.), nnquynh173@gmail.com (Q.N.N.); seoul@gachon.ac.kr (G.S.H.); norikoy@gachon.ac.kr (N.Y.); 2 Department of Food Science and Biotechnology, Kyonggi University, Suwon 16227, Korea; rhemf@kgu.ac.kr; 3 College of Bio-Nano Technology, Gachon University, Seongnam-si, Gyeonggi-do 13120, Korea; sullimlee@gachon.ac.kr

**Keywords:** *Artemisia absinthium*, shikimic acid, cisplatin, nephrotoxicity, apoptosis

## Abstract

Nephrotoxicity is a serious side effect of cisplatin, which is one of the most frequently used drugs for cancer treatment. This study aimed to assess the renoprotective effect of *Artemisia absinthium* extract and its bioactive compound (shikimic acid) against cisplatin-induced renal injury. An in vitro assay was performed in kidney tubular epithelial cells (LLC-PK1) with 50, 100, and 200 µg/mL *A. absinthium* extract and 25 and 50 µM shikimic acid, and cytotoxicity was induced by 25 µM cisplatin. BALB/c mice (6 weeks old) were injected with 16 mg/kg cisplatin once and orally administered 25 and 50 mg/kg shikimic acid daily for 4 days. The results showed that the *A. absinthium* extract reversed the decrease in renal cell viability induced by cisplatin, whereas it decreased the reactive oxidative stress accumulation and apoptosis in LLC-PK1 cells. Shikimic acid also reversed the effect on cell viability but decreased oxidative stress and apoptosis in renal cells compared with the levels in the cisplatin-treated group. Furthermore, shikimic acid protected against kidney injury in cisplatin-treated mice by reducing serum creatinine levels. The protective effect of shikimic acid against cisplatin-mediated kidney injury was confirmed by the recovery of histological kidney injury in cisplatin-treated mice. To the best of our knowledge, this study is the first report on the nephroprotective effect of *A. absinthium* extract and its mechanism of action against cisplatin-induced renal injury.

## 1. Introduction

Cis-diamminedichloroplatinum (II) was first reported as an anti-cancer agent in the 1970s and named cisplatin [1,2,3]. Clinical research has revealed that cisplatin causes renal injury in patients with cancer, which continues to limit its use [4,5,6]. Similar to many other chemotherapy drugs, cisplatin causes a wide range of adverse effects on normal tissues such as ototoxicity [7], cardiotoxicity, nephrotoxicity, hematological toxicity, gastrointestinal toxicity, hepatotoxicity, and neurotoxicity [8]. Among these side effects of cisplatin, nephrotoxicity is considered to be the most severe and to cause the most long-lasting harm to the body [9]. Moreover, nephrotoxicity is the primary reason for the discontinuation of chemotherapy in patients with cancer [10]. There are several types of nephrotoxicity that have been observed in cisplatin-treated patients. However, the most serious and frequently reported type of cisplatin nephrotoxicity is acute kidney injury, which occurs in 20–30% of patients [11]. Many clinical trials have been conducted on patients with cancer to evaluate the protective effects of potential agents against cisplatin-induced nephrotoxicity [12,13,14,15,16,17,18]. However, some of these agents were shown to have no effect, whereas others were only protective in one type of cancer, and most agents had limitations. Therefore, more studies are needed to develop additional approaches to protect the kidneys against cisplatin-induced cytotoxicity. Recently, the renoprotective effects of phytochemicals have been investigated to determine if they can prevent cisplatin-induced renal cytotoxicity [19,20,21,22,23]. Bioactive compounds from natural products represent a potential adjuvant therapy to reduce the side effects of cisplatin because they have been shown to be safe in long-term treatments and not affect the anticancer activity of cisplatin [24]. 

Among herbal medicines, *Artemisia absinthium* has been shown to have many beneficial effects in folk medicine and clinical trials [25] including nephroprotective effects [26]. *A. absinthium* is a perennial herb belonging to the *Asteraceae* family and is commonly known as wormwood. *A. absinthium* is a medicinal plant that has been recognized as an antioxidant that can be consumed as a part of the daily diet [27,28]. In previous studies, *A. absinthium* was used to provide nephroprotection against immunoglobulin A (IgA) nephropathy [29] and protect against inflammation in patients with Crohn’s disease [30,31]. Oxidative stress and apoptosis are the major mechanisms by which cisplatin-induced nephrotoxicity occurs [32,33,34]. Moreover, *A. absinthium* was reported to reduce renal toxicity caused by azathioprine treatment by regulating oxidative stress in rats [35]. *A. absinthium* as an antioxidant phytochemical may protect against cisplatin-induced nephrotoxicity [36]. Therefore, it would be of value to analyze the bioactive compounds of *A. absinthium* and investigate the mechanisms by which they protect against cisplatin-induced nephrotoxicity.

The main aim of this study was to investigate the protective effects of *A. absinthium* and its bioactive compound against cisplatin-induced kidney injury. Kidney epithelial cells (LLC-PK1) were used to examine the mechanism of this nephroprotective effect against cisplatin-induced cell damage by evaluating oxidative stress and apoptosis pathways. Indicators of cell damage in LLC-PK1 cells were evaluated, such as cell viability, reactive oxygen species (ROS) accumulation, and apoptotic ratio. Furthermore, an animal model using male BALB/c mice was established to examine the protective effects of the active compound from *A. absinthium* on the common indicators of acute kidney injury: serum creatinine level and kidney histological damage. 

## 2. Results

### 2.1. Protective Effect of A. absinthium Extract against Cisplatin-Induced Kidney Cell Death

**Figure 1:**
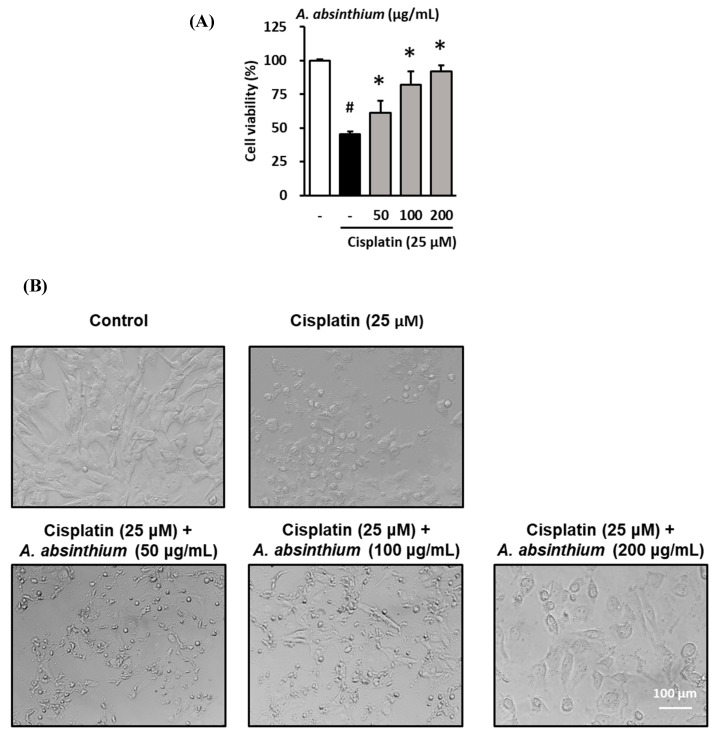
Protective effect of *A. absinthium* extract against the cisplatin-induced decrease in kidney cell viability. (**A**) Effect of the extract on the viability of LLC-PK1 cells exposed to 25 µM cisplatin for 24 h using the Ez-Cytox cell viability assay kit. (**B**) The change in morphology of LLC-PK1 cells after treatment with cisplatin and extracts. Results are the mean ± SD. The difference in the mean values between groups was assessed using the Tukey method for one-way analysis of variance (ANOVA). # *p* < 0.05 versus the control group (first column) and * *p* < 0.05 versus the cisplatin-treated group (second column). SD, standard deviation.

Kidney tubular cells (LLC-PK1) were co-treated with various concentrations of *A. absinthium* extract (50, 100, 200 µg/mL) and 25 µM cisplatin for 24 h. Cell viability was measured using the Ez-Cytox cell viability assay kit. As shown in Figure 1A, the cell viability in the cisplatin-treated group was 45.4 ± 2.08%, whereas the cell viability in the groups treated with 50, 100, and 200 µg/mL *A. absinthium* extract was 61.0 ± 9.2%, 81.9 ± 10.1%, and 91.9 ± 4.6%, respectively. These results suggest that *A. absinthium* extract has a substantial protective effect against the reduction in cell viability caused by cisplatin treatment.

Besides this, *A. absinthium* improved the cell morphology after being damaged by cisplatin cytotoxicity (Figure 1B).

### 2.2. Inhibitory Effect of A. absinthium Extract on Cisplatin-Induced ROS Accumulation in LLC-PK1 Cells

To evaluate the effect of *A. absinthium* extract on ROS accumulation in LLC-PK1 cells by cisplatin treatment, the cells were treated with 50, 100, and 200 µg/mL *A. absinthium* extract and 25 µM cisplatin for 24 h. Then, the intracellular ROS accumulation was assessed via fluorescence using 2′,7′-dichlorodihydrofluorescein diacetate (DCFDA). Fluorescence images of the cells were captured using an inverted microscope.

**Figure 2:**
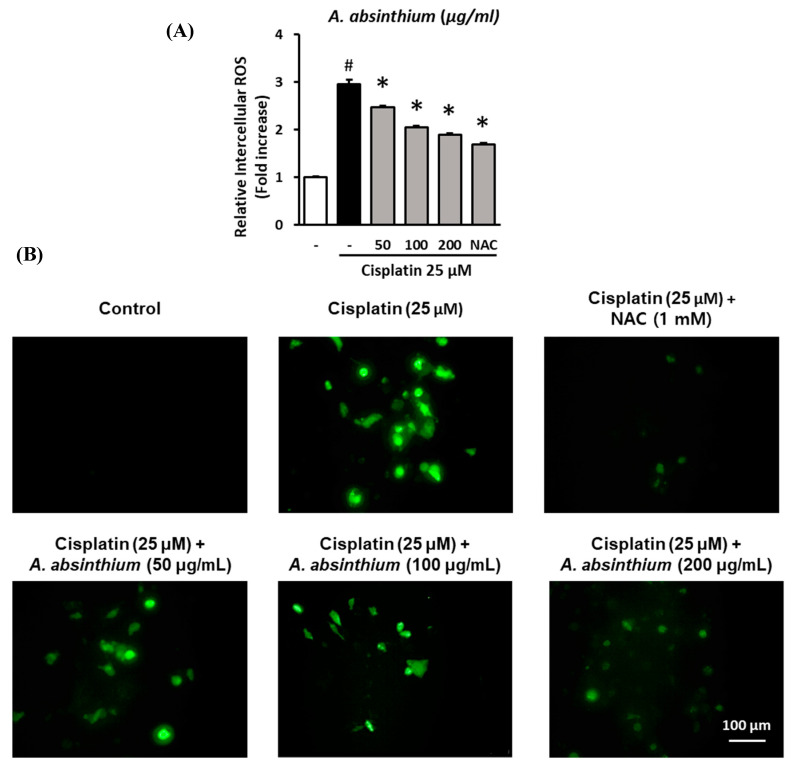
Inhibitory effect of *A. absinthium* extract on cisplatin-induced ROS accumulation in LLC-PK1 cells. LLC-PK1 cells were treated with extract and NAC 1 mM exposed 25 µM cisplatin for 24 h. Next, the intracellular ROS accumulation was assessed via fluorescence using 2′,7′-dichlorodihydrofluorescein diacetate (DCFDA). Fluorescence images of the cells were captured using an inverted microscope. (**A**) The ratio of ROS accumulation between the treated groups and untreated group. (**B**) Representative images of the fluorescence signals of intercellular ROS in LLC-PK1 cells treated with various concentrations of *A. absinthium* extract or NAC and cisplatin (Green). ROS, reactive oxygen species; DCFDA, 2′,7′-dichlorodihydrofluorescein diacetate; NAC, *N*-acetyl-l-cysteine. Results are the mean ± SD. The difference in the mean values between groups was assessed using the Tukey method for one-way analysis of variance (ANOVA). # *p* < 0.001 versus the control group (first column) and * *p* < 0.001 versus the cisplatin-treated group (second column). SD, standard deviation.

*A. absinthium* extract protected LLC-PK1 from cisplatin-mediated intercellular ROS increase. As showed in Figure 2A, ROS accumulation in the cisplatin-treated group was 2.98 ± 0.08-fold compared to the control group (*p* < 0.001), whereas the ROS level in 50 and 100 µg/mL *A. absinthium-*extract-treated group were 2.48 ± 0.03-fold and 2.06 ± 0.02-fold, respectively, lower than that in the cisplatin-treated group. Furthermore, 200 µg/mL *A. absinthium* extract and *N*-acetyl-l-cysteine (NAC) 1 mM also reduced the intercellular ROS of the cells after cisplatin treatment to 1.89 ± 0.03-fold and 1.70 ± 0.03-fold (*p* < 0.001). *A. absinthium* extract reduced the ROS accumulation of LLC-PK1 cells after cisplatin treatment in a concentration-dependent manner.

The protection of *A. absinthium* extract against ROS increase under cisplatin treatment were shown in Figure 2B by the decrease in fluorescent level of NAC and *A. absinthium-*extract-treated groups compared to cisplatin-treated group.

### 2.3. Inhibitory Effect of A. absinthium Extract on Cisplatin-Induced Apoptosis in LLC-PK1 Cells

To assess the protection of *A. absinthium* extract against cisplatin-induce apoptosis on LLC-PK1 cells. The cells were treated with various concentrations of extract (50, 100, 200 µg/mL) and exposed to 25 µM cisplatin for 24 h. Next, the cells were stained with Alexa Flour 488 annexin V conjugate, and fluorescence images of the cells were captured using a Tali Image-based Cytometer. 

**Figure 3:**
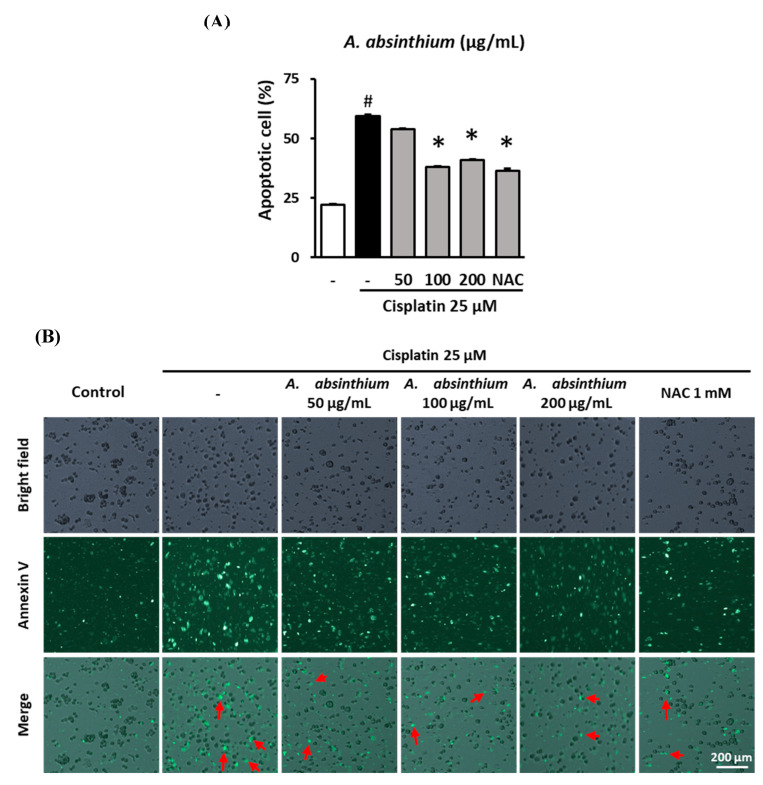
Inhibitory effect of *A. absinthium* extract on cisplatin-induced apoptosis in kidney cells. LLC-PK1 cells were treated with *A. absinthium* extract and 25 µM cisplatin for 24 h and then stained with Alexa Fluor 488 annexin V conjugate. The number of apoptotic cells was determined with TaliPCApp software. Next, fluorescence images of the cells were captured using a Tali Image-based cytometer. (**A**) The percentage of apoptotic LLC-PK1 cells between the treated groups and untreated group. (**B**) Representative images of the fluorescence signal indicating apoptosis in LLC-PK1 cells treated with various concentrations of *A. absinthium* extract or NAC and 25 µM cisplatin for 24 h using Alexa Fluor 488 annexin V conjugate staining (Green). NAC, N-acetyl-l-cysteine. Results are the mean ± SD. The difference in the mean values between groups was assessed using the Tukey method for one-way analysis of variance (ANOVA). # *p* < 0.001 versus the control group (first column) and * *p* < 0.001 versus the cisplatin-treated group (second column). SD, standard deviation. The red arrows indicate apoptotic bodies.

*A. absinthium* extract reduced the apoptosis of LLC-PK1 cells under cisplatin cytotoxicity. The percentage of apoptotic cells were significant increase in cisplatin-treated group (59.50 ± 0.46%) compared to control group (22.00 ± 0.60%) (*p* < 0.001); however, 50 and 100 µg/mL *A. absinthium-*extract-treated groups reduced the ratio of cells that undergo apoptosis to 38.00 ± 0.46% and 41.00 ± 0.22%, after cisplatin treatment, respectively (Figure 3A). As a positive control, NAC at the concentration of 1 mM decreased apoptosis level of the cells to 36.50 ± 0.82, significantly lower than that in the cisplatin-treated group (*p* < 0.001).

The decrease in apoptotic cells in the NAC- and *A. absinthium-*extract-treated groups was exhibited by the reduction in the fluorescence signal compared with cisplatin-treated group (Figure 3B, Merged pictures). 

### 2.4. High-Performance Liquid Chromatography (HPLC) of A. absinthium Extract

**Figure 4:**
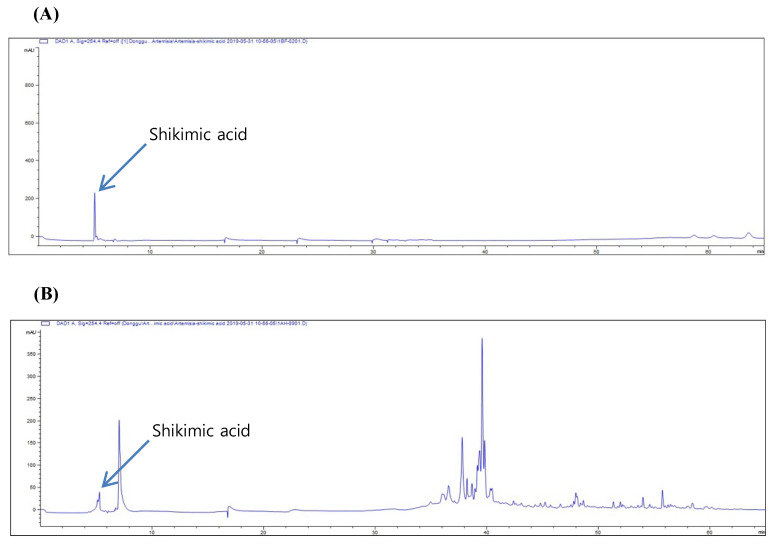
HPLC chromatograms of standard shikimic acid (**A**) and HPLC chromatograms of *A. absinthium* extract (**B**).

To determine the bioactive compounds in *A. absinthium* extract, HPLC was conducted, and shikimic acid (standard compound) was analyzed according to retention time. As shown in Figure 4A, the quantification parameter for shikimic acid was examined using the aforementioned HPLC conditions. Further, the calibration curves for shikimic acid that were constructed by plotting the peak areas of prepared concentrations were found to be linear when evaluated using linear regression analysis (Figure 4A). Linearity was studied using six solutions in the concentration range 0.62–1.00 mg/mL (*n* = 6). The regression equation was determined by plotting the peak area (y) versus the shikimic acid concentration (x) expressed in mg/mL. The correlation coefficient (*r*^2^ = 0.998) obtained for the regression line indicates that there is a strong linear relationship between the peak area and concentration of shikimic acid (Figure 4B). The yield of shikimic acid from *A. absinthium* was 2.277 ± 0.145 mg/g.

### 2.5. Protective Effect of Shikimic Acid against Cisplatin-Induced Kidney Cell Damage

To assess the protective effect of shikimic acid against cisplatin-mediated kidney cell death, LLC-PK1 cells were treated with several concentrations of shikimic acid (6.125, 12.5, 25, 50, and 100 µM), and cytotoxicity was induced by treatment with 25 µM cisplatin for 24 h. NAC was used as a positive control at concentrations of 100 and 1000 µM. Cell viability was measured using the Ez-Cytox cell viability assay kit.

**Figure 5:**
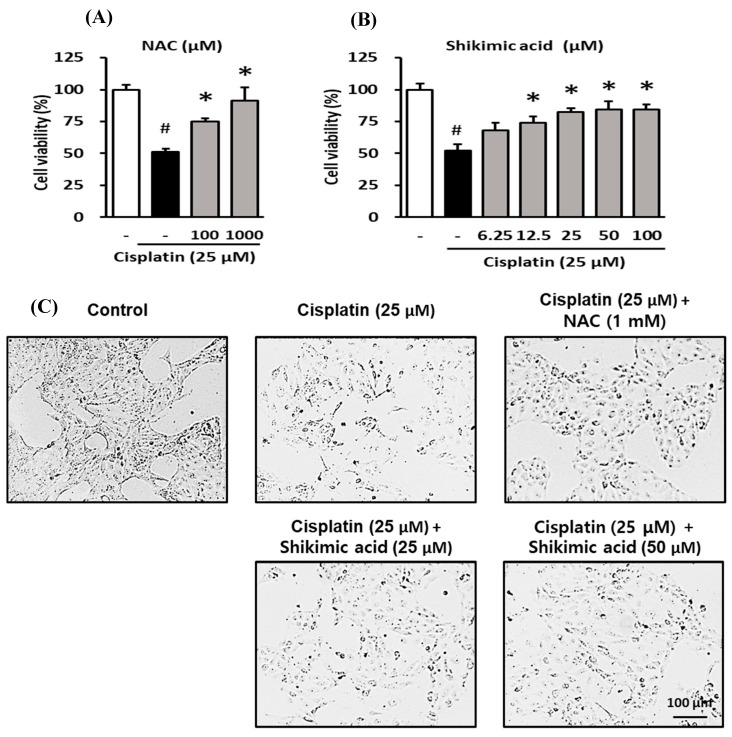
Protective effect of shikimic acid against cisplatin-induced kidney cell damage. The cell viability of LLC-PK1 cell was assessed using an Ez-Cytox cell viability assay kit, and the morphological changes in the cells were assessed using an inverted microscope. (**A**) Effect of NAC on the viability of LLC-PK1 cells exposed to 25 µM cisplatin for 24 h. (**B**) Effect of shikimic acid on the viability of LLC-PK1 cells exposed to 25 µM cisplatin for 24 h. (**C**) Effect of NAC and shikimic acid on cell density and morphology changes in LLC-PK1 cells exposed to 25 µM cisplatin for 24 h. Results are the mean ± SD. The difference in the mean values between groups was assessed using the Tukey method for one-way analysis of variance (ANOVA). # *p* < 0.001 versus the control group (first column) and * *p* < 0.001 versus the cisplatin-treated group (second column). NAC, *N*-acetyl-l-cysteine; SD, standard deviation.

NAC ameliorated the reduction in the viability of LLC-PK1 cells induced by cisplatin treatment. As shown in Figure 5A, the viability of LLC-PK1 cells decreased to 52.2% in the cisplatin-treated group and recovered to 74.8 ± 2.88% and 91.4 ± 10.28% in the 100 and 1000 µM NAC groups, respectively. 

Similarly, shikimic acid protected LLC-PK1 cells against the cisplatin-induced decrease in cell viability. The cell viability in the cisplatin-treated group decreased significantly to 52.2 ± 4.71% compared to that in the control group (*p* < 0.05) (Figure 5B). Meanwhile, the cell viability in the shikimic-acid-treated groups recovered to 68.3 ± 5.75% and 74.1 ± 4.95% at 6.25 and 12.5 µM, respectively. Additionally, the cell viability in 25, 50, and 100 µM shikimic-acid-treated groups increased to 82.3 ± 3.03%, 84.5 ± 6.15%, and 84.4 ± 3.91%, respectively (Figure 5B). In the groups treated with shikimic acid at concentrations of 12.5 µM and higher, cell viability increased significantly compared with that in the cisplatin-treated group (*p*-value < 0.05). Comparing the results shown in Figure 5A,B, the recovered cell viability in the 25 and 50 µM shikimic acid groups (84.5% and 84.4%) was higher than that in the 100 µM NAC group (74.8%). Therefore, shikimic acid concentrations of 25 and 50 µM were used in subsequent experiments.

Shikimic acid also protected against the cisplatin-induced effects on cell density decrease and morphological changes in LLC-PK1 cells. As shown in Figure 5C, cell density in the cisplatin-treated group was lower than that in the control group. However, the cell densities in the shikimic-acid-treated groups (25 and 50 µM) and the NAC (1000 µM) positive control group were higher than that in the cisplatin-treated group. Moreover, the morphology of LLC-PK1 cells was damaged in the cisplatin-treated group compared with that in the control group. However, the damaged morphology of LLC-PK1 cells caused by cisplatin cytotoxicity recovered in the NAC- and shikimic-acid-treated groups.

These data indicate that shikimic acid reduced the damage to LLC-PK1 cells caused by cisplatin cytotoxicity. In fact, shikimic acid induced recovery from the effects on cell density and morphology, leading to increased cell survival. The extent of this recovery can be estimated by comparison with the positive control groups treated with NAC at 100 and 1000 µM.

### 2.6. Inhibitory Effect of Shikimic Acid on Cisplatin-Induced ROS Accumulation in LLC-PK1 Cells

To evaluate the effect of shikimic acid on ROS accumulation in LLC-PK1 cells under cisplatin treatment, the cells were treated with 25 or 50 µM shikimic acid and 25 µM cisplatin for 24 h. Following this treatment, the intracellular ROS accumulation was assessed via fluorescence using 2′,7′-dichlorodihydrofluorescein diacetate (DCFDA). Fluorescence images of the cells were captured using an inverted microscope.

**Figure 6:**
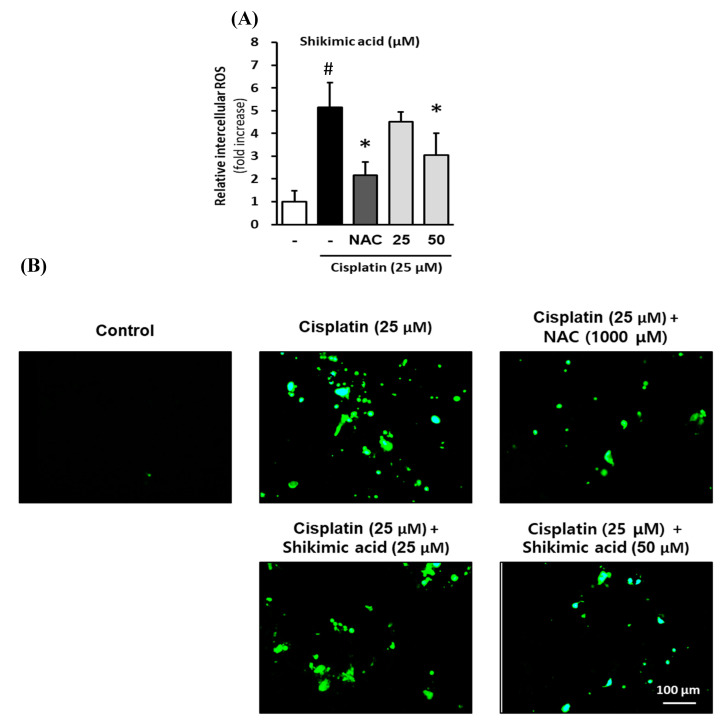
Inhibitory effect of shikimic acid on cisplatin-induced ROS accumulation in LLC-PK1 cells. LLC-PK1 cells were treated with 25 or 50 µM shikimic acid and 25 µM cisplatin for 24 h. Next, the intracellular ROS accumulation was assessed via fluorescence using DCFDA. Fluorescence images of the cells were captured using an inverted microscope. (**A**) The ratio of ROS accumulation between the treated groups and untreated group. (**B**) Representative images of the fluorescence signals of intercellular ROS in LLC-PK1 cells treated with various concentrations of shikimic acid or NAC and cisplatin (Green). ROS, reactive oxygen species; DCFDA, 2′,7′-dichlorodihydrofluorescen diacetate; NAC, *N*-acetyl-l-cysteine. Results are the mean ± SD. The difference in the mean values between groups was assessed using the Tukey method for one-way analysis of variance (ANOVA). # *p* < 0.001 versus the control group (first column) and * *p* < 0.001 versus the cisplatin-treated group (second column). SD, standard deviation.

Shikimic acid protected LLC-PK1 cells against the cisplatin-induced increase in intercellular ROS. As shown in Figure 6A, ROS accumulation in the cisplatin-treated group was 5.14 ± 1.10-fold higher than that in the control group *(p* < 0.001), whereas the intracellular ROS level in the 1000 µM NAC-treated group was 2.17 ± 0.57-fold higher than that in the control group. In addition, ROS accumulation in the 25 and 50 µM shikimic acid groups was 4.50 ± 0.45-fold and 3.05 ± 0.96-fold, respectively, higher than that in the control group. However, the ratio of intracellular ROS in the NAC- and shikimic-acid-treated (50 µM) groups was significantly lower than that in the cisplatin-treated group *(p* < 0.05).

The effect of shikimic acid on cisplatin-induced ROS accumulation in LLC-PK1 cells was confirmed by the decrease in the fluorescence signal (Figure 6B). The fluorescence signal in the cisplatin-treated group was higher than that in the control group, indicating that ROS accumulation in LLC-PK1 cells in the cisplatin-treated group was higher than that in the control group. However, the fluorescence levels in the 1000 µM NAC and shikimic-acid-treated group (50 µM) were lower than that in the cisplatin-treated group. Therefore, the intercellular ROS levels in the NAC- and shikimic-acid-treated groups decreased compared with that in the cisplatin-treated group. These results support the hypothesis that shikimic acid inhibits oxidative stress in kidney cells following cisplatin treatment by reducing cellular ROS accumulation.

### 2.7. Inhibitory Effect of Shikimic Acid on Cisplatin-Induced Apoptosis in LLC-PK1 Cells

To determine whether shikimic acid protected kidney cells from apoptosis induced by cisplatin toxicity, LLC-PK1 cells were treated with 25 or 50 µM shikimic acid and 25 µM cisplatin for 24 h. Cells were then stained with Alexa Fluor 488 annexin V conjugate, and fluorescence images of the cells were captured using a Tali Image-based Cytometer.

**Figure 7:**
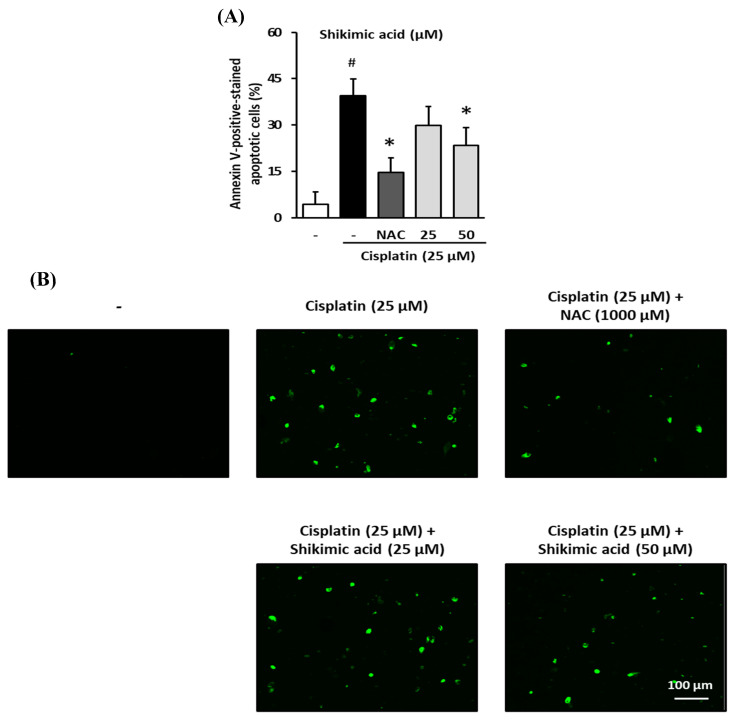
Inhibitory effect of shikimic acid on cisplatin-induced apoptosis in kidney cells. LLC-PK1 cells were treated with 25 or 50 µM shikimic acid and 25 µM cisplatin for 24 h and then stained with Alexa Fluor 488 annexin V conjugate. The number of apoptotic cells was determined with TaliPCApp software. Next, fluorescence images of the cells were captured using a Tali Image-based cytometer. (**A**) The percentage of apoptotic LLC-PK1 cells between the treated groups and untreated group. (**B**) Representative images of the fluorescence signal indicating apoptosis in LLC-PK1 cells treated with various concentrations of shikimic acid or NAC and 25 µM cisplatin for 24 h using Alexa Fluor 488 annexin V conjugate staining (Green). Results are the mean ± SD. NAC, N-acetyl-l-cysteine. The difference in the mean values between groups was assessed using the Tukey method for one-way analysis of variance (ANOVA). # *p* < 0.001 versus the control group (first column) and * *p* < 0.001 versus the cisplatin-treated group (second column). SD, standard deviation.

Shikimic acid exerted a protective effect against the cisplatin-induced increase in apoptosis in LLC-PK1 cells. The proportion of apoptotic cells in the cisplatin-treated group (39.4 ± 5.56%) was significantly higher than that in the control group (4.4%; *p* < 0.05) (Figure 7A). However, the percentage of apoptotic cells in the NAC group (1000 µM) decreased to 14.6 ± 4.86%, whereas the percentages in the shikimic-acid-treated groups (25 and 50 µM) decreased to 29.8 ± 6.22% and 23.4 ± 5.83, respectively. Indeed, the percentage of apoptotic cells in the NAC- and shikimic-acid-treated (50 µM) groups was significantly lower than that in the cisplatin-treated group (*p* < 0.05).

The decrease in apoptotic cells in the NAC- and shikimic-acid-treated groups was evidenced by the reduction in the fluorescence signal (Figure 7B). As seen in a representative image from the cisplatin group, there was a significant increase in fluorescence compared to that in the control group, indicating an increase in the number of apoptotic cells. However, compared with that in the cisplatin-treated group, the fluorescence signals in the 1000 µM NAC and shikimic-acid-treated (50 µM) groups were lower; therefore, NAC and shikimic acid reduced the number of apoptotic cells after cisplatin treatment. These results indicate that shikimic acid protected LLC-PK1 cells from apoptosis induced by cisplatin. 

### 2.8. Effect of Shikimic Acid on the Cisplatin-Induced Increase in Serum Creatinine and Kidney Injury in BALB/c Mice 

To evaluate the effect of shikimic acid on the increase in serum creatinine induced by cisplatin nephrotoxicity, a mouse model of cisplatin-induced acute kidney injury was established, and the serum creatinine levels in the mice were measured. Next, the kidneys were extracted, fixed in formalin, and stained with hematoxylin & eosin (H&E) for histological examination.

**Figure 8:**
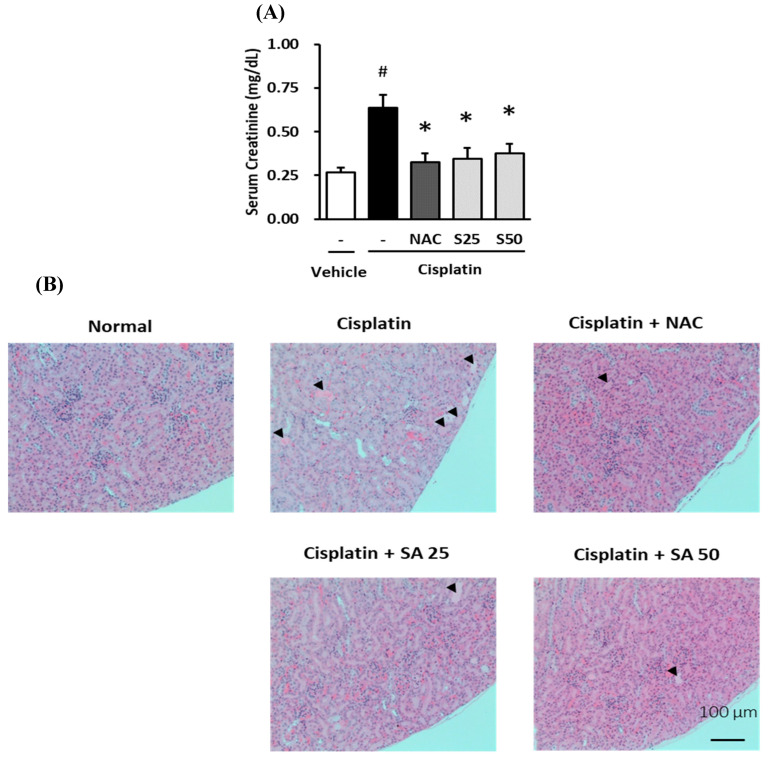
Protective effect of shikimic acid against the cisplatin-induced serum creatinine increase and histological kidney injury in 6-week-old BALB/c mice. Mice were injected with physiological saline (normal) or 16 mg/kg cisplatin (cisplatin) in physiological saline before being treated with NAC (1000 mg/kg) or shikimic acid (25 mg/kg, SA25, and 50 mg/kg, SA50) (*n* = 5–6 mice/group) for 4 d. Serum creatinine was measured using an AceChem Creatinine kit. (**A**) Serum creatinine levels in mice treated with shikimic acid or NAC after being injected with cisplatin. (**B**) Hematoxylin & Eosin (H&E) staining of representative kidney sections of the shikimic-acid- and NAC-treated mice after being injected with cisplatin. Black arrows indicate an area of kidney injury. Results are the mean ± SEM. NAC, N-acetyl-l-cysteine; SA, shikimic acid; SEM, standard error of the mean; H&E, hematoxylin & eosin. The difference in the mean values between groups was assessed using the Tukey method for one-way analysis of variance (ANOVA). # *p* < 0.001 versus the control group (first column) and * *p* < 0.05 versus the cisplatin-treated group (second column).

Shikimic acid reduced the increase in the serum creatinine level in mice caused by cisplatin treatment. As shown in Figure 8A, the serum creatinine level in the cisplatin-treated group increased significantly to 0.64 ± 0.07 mg/dL compared with that in the normal group (0.27 ± 0.3 mg/dL; *p* = 0.0007). By contrast, the serum creatinine level in the 1000 mg/kg NAC-treated group was 0.32 ± 0.05 mg/dL, which was significantly lower than that in the cisplatin-treated group (*p* = 0.004). In addition, the serum creatinine levels in the 25 mg/kg shikimic-acid-treated group (0.34 ± 0.06; *p* = 0.0186) and the 50 mg/kg shikimic-acid-treated group (0.38 ± 0.06 mg/dL; *p* = 0.0194) were significantly lower than that in the cisplatin-treated group. Furthermore, the serum creatinine levels in the shikimic-acid-treated groups (25 and 50 mg/kg) recovered to a level similar to that in the normal group (*p* = 0.902 and *p* = 0.633, respectively). These results indicate that there was no statistical difference in serum creatinine levels between the shikimic-acid-treated groups and the control group. In other words, shikimic acid caused the recovery of the serum creatinine level in mice to normal levels after cisplatin treatment.

Shikimic acid ameliorated the kidney injury in mice treated with cisplatin. As shown in the images, the kidneys in the cisplatin-treated group were damaged compared with those in the normal group (Figure 8B). However, the kidney sections in the NAC- and shikimic-acid-treated groups indicated recovery from the injury induced by cisplatin. These data indicate that shikimic acid reduced serum creatinine levels and ameliorated cisplatin-induced kidney damage.

## 3. Discussion

Nephrotoxicity is one of the major side effects of cisplatin, which limits its use in cancer treatment [37]. Therefore, finding the adjuvant agents to reduce cisplatin nephrotoxicity is an important approach for cisplatin cancer therapeutic. Our previous studies had reported other agents from natural products which had potential in reducing cisplatin nephrotoxicity [38,39,40]. In this study, we focused on the renoprotective effect of shikimic acid isolated from *A. absinthium* on LLC-PK1 cells and mouse model. A previous study reported that oxidative stress and apoptosis were two of the three main pathological mechanisms of cisplatin-induced nephrotoxicity [41]. NAC has long been used for chemoprotection against cisplatin toxicity in vitro, in vivo, and in clinical trials [42,43,44]; thus, we used NAC as a positive control for all experiments. Supporting our hypothesis, the percentage of cell viability in the cisplatin-treated group was lower than that in the control group. In contrast, the percentage in the *A. absinthium*-extract-treated groups recovered to a level significantly higher than that in the cisplatin-treated group. These results indicate that *A. absinthium* extract reversed the decrease in the viability of kidney cells induced by cisplatin toxicity. 

Furthermore, our data showed that *A. absinthium* extract reduced ROS accumulation and apoptotic cells death by cisplatin. In other words, *A. absinthium* extract protected LLC-PK1 cells against cisplatin-induced cytotoxicity by regulating accumulation of intracellular ROS and apoptosis pathway. Other studies have also documented that *A. absinthium* extract improved kidney dysfunction in rats with diabetes induced by alloxan administration [45] and protected the kidney from lead-induced toxicity in a rat model [46]. Ali et al. claimed that *A. absinthium* protected DNA against H_2_O_2_-induced oxidative stress in the pUC19 plasmid vector [47]. Indeed, DNA damage is the hallmark of the anticancer effect of cisplatin [48,49]. Hence, *A. absinthium* could be a potent adjuvant to reduce cisplatin-induced nephrotoxicity. *A. absinthium* had been reported as antioxidant agent [50,51] whereas other studies showed that *A. absinthium* extracts also exerted to induce oxidative stress and apoptosis in cancer cells [52,53,54,55]. Therefore, it is necessary to determine the bioactive compound in *A. absinthium* extract that has the nephroprotective effect under cisplatin treatment.

The antioxidant effect of *A. absinthium* extract has been correlated with its total phenolic content [56], whereas other studies have documented that shikimic acid was the most abundant precursor for the metabolism of phenolic compounds in plants [57,58]. In this study, we focused on analyzing the concentration of shikimic acid from *A. absinthium* extract; thus, the HPLC results were analyzed to compare the extract with shikimic acid (standard compound) according to retention time. The data showed that the amount of shikimic acid in *A. absinthium* was not as high as that reported in an earlier study in other plants [59]. However, another study claimed that shikimic acid was one of the most abundant bioactive compounds among the seven bioactive compounds in *A. absinthium* [60]. Although shikimic acid was not found to be a major compound in *A. absinthium,* shikimic acid may play a main role in the nephroprotective effect of *A. absinthium* L. extract against cisplatin cytotoxicity.

Cell damage is the first visible sign of cisplatin cytotoxicity. Moreover, cell viability has been used as an indicator to assess the damage caused by cisplatin-mediated nephrotoxicity [61]. In this study, cisplatin-induced cytotoxicity of kidney cells was assessed by measuring the cell viability of kidney tubular cells; representative images confirmed the morphological changes in LLC-PK1 cells. The data from our study showed that the cell viability in the cisplatin-treated group decreased significantly compared to that in the control group. In contrast, the cell viability in the shikimic-acid-treated groups was higher than that in the cisplatin-treated group. These results indicated that shikimic acid recovered the viability of kidney cells after treatment with cisplatin. Moreover, cell viability of the shikimic-acid-treated groups (25 and 50 µM) was higher than that in the 100 µM NAC-treated group. These results indicate that a lower concentration of shikimic acid had a more potent effect than NAC. Furthermore, cell density in the shikimic-acid-treated groups increased, and the cell morphology of these groups also recovered from cisplatin toxicity. Therefore, shikimic acid had a strong protective effect against cisplatin-mediated kidney cell damage than the positive control NAC. Manna et al. reported that shikimic acid, as an active component of coconut water, had a protective effect against hydroperoxide-induced oxidative stress in murine hepatocytes [62]. However, there have been no investigations on the protective effect of *A. absinthium* extract and its active compound, shikimic acid, against cisplatin-mediated cell damage in kidney cells.

Recent studies have clarified the complex and multiple mechanisms of cisplatin nephrotoxicity, among which oxidative stress was defined as the main pathway and the key to prevent cell death caused by cisplatin [63,64,65,66,67]. In this study, the intercellular ROS level in the cisplatin-treated group increased five-fold compared with that in the control group. However, ROS accumulation in the shikimic-acid- and NAC-treated groups was lower than that in the cisplatin-treated group. These data indicated that shikimic acid (50 µM) significantly reduced the oxidative stress in kidney cells induced by cisplatin. Shikimic acid has been reported to exert an antioxidant effect at 10 mM on the neuroblastoma SH-SY5Y cell line [68]. In the present study, the concentration of shikimic acid that produced the best antioxidant activity was 50 µM in kidney tubular LLC-PK1 cells. Hence, this result indicates a more effective response at reducing oxidative stress induced by cisplatin nephrotoxicity than the previously reported antioxidant effect against H_2_O_2_-induced toxicity in SH-SY5Y cells. This study is also the first to report the antioxidant effect of shikimic acid in kidney tubular cells (LLC-PK1).

Apoptosis has also been determined to be an essential mechanism of cell death induced by cisplatin, interfering with its anti-cancer effect [69]. Preventing apoptosis in kidney tubular cells is also an important strategy to reduce the nephrotoxicity of cisplatin [70]. As can be seen in the results, the percentage of apoptotic cells in the cisplatin-treated group increased almost ten-fold compared with that in the control group. In contrast, the proportion in the shikimic-acid- and NAC-treated groups was significantly lower than that in the cisplatin-treated group. These results indicate that shikimic acid protected kidney cells from cisplatin-mediated apoptosis. Although a high dose of cisplatin will lead to necrosis in kidney cells, a low dose will result in apoptosis [69,71]. In this study, we used cisplatin to assess the protective effect of shikimic acid against apoptosis induced by cisplatin in kidney cells (LLC-PK1). An analog of shikimic acid, 3,4-oxo-isopropylidene-shikimic acid, was found to have an anti-neuronal apoptosis effect under hypoxic conditions at its most effective concentration of 10 mg/kg in a rat model [72]. Another study showed that shikimic acid inhibited osteoclastogenesis and bone resorption in bone marrow monocytes in vitro by suppressing nuclear factor kappa-light-chain-enhancer of activated B cells and mitogen-activated protein kinase [73]. Therefore, shikimic acid may play an important role in regulating many cell death pathways. However, no previous study has reported the anti-apoptotic activity of shikimic acid in LLC-PK1 cells following cisplatin treatment.

Several studies have shown that acute kidney injury (AKI) is one of the most common and severe side effects of cisplatin in patients with cancer, having been reported in 30% of children in the early stage of cisplatin infusion [74] and in 61% during the therapy [75]. Hence, we used a high dose (16 mg/kg) of cisplatin and a short (4 d) experimental period to induce AKI in a mouse model [76]. Clinically, serum creatinine is the first measured indicator of AKI [74,75,77], and kidney injury can be assessed using histological staining [78]. Therefore, we used serum creatinine levels and histological evaluations to assess the degree of injury in this study. The serum creatinine level in the cisplatin-treated group was significantly higher than that in the normal group. In contrast, the serum creatinine levels in the NAC- and shikimic-acid-treated groups were significantly lower than that in the cisplatin-treated group. These data indicate that shikimic acid reduced the serum creatinine level in mice after treatment with cisplatin. In a previous study, a low dose of cisplatin (2.5 mg/kg once a week for 3 weeks) was administered to rats, and the serum creatinine level significantly increased after day 6 of the experiment [79]. In addition, in another study in which a high dose of cisplatin (10 mg/kg single dose) was administered, the serum creatinine level significantly increased after day 3 [80]. However, our data indicate that the serum creatinine level markedly increased on day 4 after cisplatin injection. Therefore, the results of our study agreed with those of the second study that used a high dose of cisplatin (10 mg/kg). To our knowledge, our study is the first to report the effect of shikimic acid on reducing serum creatinine levels in mice following a high dose of cisplatin.

Representative mouse kidney sections were stained with H&E to evaluate the injury caused by cisplatin toxicity. The histological images of the kidney sections indicated recovery of the renal tubules in the shikimic-acid- and NAC-treated groups compared with observations in the cisplatin-treated group. This finding was consistent with that of an earlier study in rats administered a high dose of cisplatin (10 mg/kg), which claimed that histological injury in the kidneys was recognized on day 1 and confirmed on day 3 after cisplatin treatment [80]. This investigation was in line with the results from the present study on tubular kidney LLC-PK1 cells, indicating that shikimic acid had a renoprotective effect against cisplatin-induced renal injury. 

This study has several limitations, including that the adjuvant compound may reduce not only the nephrotoxicity of cisplatin but also its anticancer activity [81]. Our study only assessed the protective effect of shikimic acid on cisplatin-mediated kidney injury without testing whether shikimic acid affected the anticancer activity of cisplatin on cancer cells. However, a previous study reported that complexes of shikimic acid with cisplatin still had anticancer effects on mouse lymphocytic leukemia cells (L1210), mouse leukemia cells (P388), and mouse melanoma cells (B16) in vivo [82]. Therefore, shikimic acid may not have affected the anticancer activity of cisplatin in this combined treatment. In summary, further studies on the interaction between shikimic acid and the anticancer effect of cisplatin are needed.

## 4. Materials and Methods 

### 4.1. Plant Extracts, Reagents and HPLC

Dried sample of *A. absinthium* (15 g) were extracted with ethanol under reflux system. This extract was dissolved in methanol and filtered with a syringe filter (0.45-μm). Chromatographic analysis was performed using an HPLC system (Agilent Technology 1290 Infinity II, MA, USA) equipped with a pump, auto-sampler, and UV detector with an INNO C18 column (4.6 mm × 25 cm, 5 μm). A shikimic acid standard was purchased from the Natural Product Institute of Science and Technology (Anseong, Korea). HPLC-grade solvents (methanol, water, and acetonitrile) were purchased from J. T. Baker (Phillipsburg, PA, USA). Acetic acid (99.7%) was purchased from Samchun Pure Chemicals (Pyeongtaek, Korea). Gradient elution was performed using 0.5% acetic acid in water (A) and acetonitrile (B) at a flow rate of 1 mL/min. The initial condition was set at 95% (A) and decreased to 90% (A) after 20 min. Solvent A was further decreased to 50% until 40 min, then to 10% until 50 min, and maintained for 10 min. It was increased from 0% to 95% until 65 min. The total analysis time was 65 min. The flow rate of the mobile phase was 1 mL/min. The injection volume was 10 μL, and the detector was set at a UV absorbance of 254 nm. The column temperature was maintained at 30 °C. 

### 4.2. Assessment of the Effect of A. absinthium Extract and Shikimic Acid on Cisplatin-Induced Nephrotoxicity 

#### 4.2.1. Cell Culture and Treatment

The LLC-PK1 kidney tubular cell line (ATCC, Bethesda, MD, USA) was cultured in Dulbecco’s Modified Eagle Medium (DMEM, Corning, Manassas, VA, USA) supplemented with 10% fetal bovine serum (FBS, Gibco BRL, Carlsbad, MD, USA) and 1% penicillin-streptomycin solution (Life Technologies, Waltham, MA, USA). The cells were maintained in a humid atmosphere of 5% CO_2_ and 37 °C.

#### 4.2.2. Evaluation of the Protective Effects of A. absinthium Extract and Shikimic Acid against Kidney Cell Damage

The effects of *A. absinthium* extract and shikimic acid on the viability of LLC-PK1 cells were measured using the Ez-Cytox cell viability assay kit (Daeil Lab Service Co., Seoul, Korea) [83]. LLC-PK1 cells were seeded in 96-well plates at a density of 1 × 10^4^ cells in 100 µL per well and incubated for 24 h in a humid atmosphere (5% CO_2_ and 37 °C). *A. absinthium* extract or shikimic acid was added at different doses with or without 25 µM cisplatin. The vehicle, dimethyl sulfoxide (DMSO, Sigma-Aldrich, St. Louis, MO, USA), was used as a control, and *N*-Acetyl-l-cysteine (NAC, Sigma-Aldrich, St. Louis, MO, USA) was used as a positive control. After a 24-h incubation, 10% Ez-Cytox solution in DMEM was added followed by incubation for 1 h. Absorbance was measured at 450 nm using a Spark 10M system (Tecan Group Ltd., Männedorf, Switzerland). The protective effect was calculated as a percentage of the control group. The morphological changes in the cells were assessed using an Olympus IX51 microscope (Olympus, Tokyo, Japan).

#### 4.2.3. Assessment of Intracellular ROS 

The cells were stained with 2′,7′- dichlorodihydrofluorescein diacetate (DCFDA, Sigma-Aldrich, St. Louis, MO, USA), and then the fluorescence intensity of DCFDA was measured [84]. LLC-PK1 cells were seeded in 96-well black plates at a density of 1 × 10^4^ cells per well for 24 h. The cells were then treated with shikimic acid or *A. absinthium* extract at various concentrations with or without 25 µM cisplatin. DMSO was used as a control, and NAC was used as a positive control. After 24 h, the cells were stained with DCFDA for 30 min, followed by three washes with Dulbecco’s phosphate-buffered saline (DPBS, Welgene Inc., Daegu, Korea). The fluorescence was measured at am excitation wavelength of 480 nm and an emission wavelength of 530 nm using a SPARK 10M system. ROS accumulation was calculated as the percentage of the control group. Fluorescence images of the cells were captured using an inverted microscope (Olympus IX51).

#### 4.2.4. Evaluation of Apoptotic Cells 

Apoptotic cells were assessed using a cytometric assay kit (Invitrogen, Temecula, CA, USA) to confirm the protective effect of *A. absinthium* extract or shikimic acid against cisplatin-induced apoptosis [85]. LLC-PK1 cells were seeded in 6-well plates at a density of 1 × 10^6^ cells in 2 mL per well for 24 h (5% CO_2_ and 37 °C). The cells were then treated with *A. absinthium* extract or shikimic acid at various concentrations with or without 25 µM cisplatin. DMSO was used as a control, and NAC was used as a positive control. After 24 h, the cells were harvested and stained with Alexa Fluor 488-conjugated annexin V (Invitrogen, Temecula, CA, USA) for 20 min in the dark, followed by washing with DPBS. Fluorescence images of the cells were captured using a Tali Image-based Cytometer (Invitrogen, Temecula, CA, USA). The number of apoptotic cells was determined using TaliPCApp software (version 1.0). The results were calculated as the percentage of cells stained with annexin V versus the total number of cells in each group.

### 4.3. Assessment of the Renoprotective Effect of Shikimic Acid against Cisplatin-Mediated Renal Injury in Male Mice

Six-week-old male BALB/c mice were used to assess the renoprotective effect of shikimic acid against cisplatin-induced renal injury (GIACUC-R2019026 approved 7 October 2019). Cisplatin in physiological saline was administered to mice through one intraperitoneal (IP) administration at a dose of 16 mg/kg. From the day of IP administration of cisplatin, a daily dose of 25 and 50 mg/kg shikimic acid was administered orally for 4 days. As positive and negative controls, NAC (1000 mg/kg) and saline, respectively, were orally administered to normal mice [86]. Four days after the IP administration of cisplatin, the mice were anesthetized with diethyl ether, and blood was collected. Creatinine levels in the blood were measured using an AceChem Creatinine Kit according to the manufacturer’s instructions (Yeongdong Pharmaceutical Co., Ltd., Chungcheongbuk, Korea). Next, the absorbance was measured using a SPARK 10M system. Moreover, to evaluate the recovery from histological kidney injury in cisplatin-treated mice, extracted kidneys were fixed in formalin and stained with hematoxylin and eosin (H&E). The serum creatinine level was calculated as a percentage of the control group, and the recovery from the histological kidney injury was compared with that in cisplatin-treated mice.

### 4.4. Statistical Analysis

All experiments were independently performed at least in triplicate. The data are presented as the mean ± SD (standard deviation) for cell experiments and mean ± SEM (standard error of mean) for animal experiments. The difference in the mean values between groups was assessed using the Tukey method for one-way analysis of variance (ANOVA) using R statistical software (version 3.3.3) [87]. A *p*-value of less than 0.05 or 0.001 was considered statistically significant.

## 5. Conclusion

In conclusion, *A. absinthium* extract increased the viability, whereas it decreased ROS accumulation and apoptotic kidney cell damages by cisplatin, and shikimic acid exerted a protective effect against cisplatin-induced nephrotoxicity both in vivo and in vitro. The protective effect of *A. absinthium* extract and shikimic acid against cisplatin cytotoxicity in kidney cells occurred through oxidative stress and apoptosis pathways. *A. absinthium* extract and shikimic acid regulated cisplatin-induced oxidative stress in LLC-PK1 cells by reducing the accumulation of ROS, a product of oxidative stress. Furthermore, *A. absinthium* extract and shikimic acid ameliorated apoptosis, which is one of the hallmarks of cisplatin cytotoxicity, in cisplatin-treated kidney cells. Shikimic acid also reversed the increase in serum creatinine levels and induced recovery of the histological injury of mouse kidneys following treatment with cisplatin. Therefore, *A. absinthium* extract and shikimic acid represent potential therapeutic approaches for reducing or preventing cisplatin nephrotoxicity. The results of this study add to the knowledge of the bioactivity of *A. absinthium* extract and revealed a new pharmacological effect of shikimic acid.

## References

[1] Rosenberg B.H., Vancamp L., Trosko J.E., Mansour V.H. (1969). Platinum Compounds: A New Class of Potent Antitumour Agents. Nat. Cell Biol..

[2] Rosenberg B., Vancamp L. (1970). The successful regression of large solid sarcoma 180 tumors by platinum compounds. Cancer Res..

[3] Higby D.J., Wallace H.J., Albert D., Holland J. (1974). Diaminodichloroplatinum: A phase I study showing responses in testicular and other tumors. Cancer.

[4] Erdlenbruch B., Pekrum A., Roth C., Grunewald R.W., Kern W., Lakomek M. (2001). Cisplatin nephrotoxicity in children after continuous 72-h and 3x1-h infusions. Pediatr. Nephrol..

[5] Leu L., Baribeault D. (2009). A comparison of the rates of cisplatin (cDDP)-induced nephrotoxicity associated with sodium loading or sodium loading with forced diuresis as a preventative measure. J. Oncol. Pharm. Pract..

[6] Duan Z., Cai G., Li J., Chen X. (2020). Cisplatin-induced renal toxicity in elderly people. Ther. Adv. Med. Oncol..

[7] Bokemeyer C., Berger C., Hartmann J., Kollmannsberger C., Schmoll H., Kuczyk M., Kanz L. (1998). Analysis of risk factors for cisplatin-induced ototoxicity in patients with testicular cancer. Br. J. Cancer.

[8] Oun R., Moussa Y.E., Wheate N.J. (2018). The side effects of platinum-based chemotherapy drugs: A review for chemists. Dalton Trans..

[9] Latcha S., Jaimes E.A., Patil S., Glezerman I.G., Mehta S., Flombaum C.D. (2016). Long-Term Renal Outcomes after Cisplatin Treatment. Clin. J. Am. Soc. Nephrol..

[10] Hoek J., Bloemendal K.M., Van Der Velden L.-A.A., Van Diessen J., Van Werkhoven E., Klop W.M.C., Tesselaar M.E.T. (2016). Nephrotoxicity as a Dose-Limiting Factor in a High-Dose Cisplatin-Based Chemoradiotherapy Regimen for Head and Neck Carcinomas. Cancers.

[11] Miller R.P., Tadagavadi R.K., Ramesh G., Reeves W.B. (2010). Mechanisms of Cisplatin Nephrotoxicity. Toxins.

[12] Uozumi J., Koikawa Y., Yasumasu T., Tokuda N., Kumazawa J. (1996). The Protective Effect of Methylprednisolone against Cisplatin-Induced Nephrotoxicity in Patients with Urothelial Tumors. Int. J. Urol..

[13] Baek S.H., Kim S.H., Kim J.W., Kim Y.J., Lee K.-W., Na K.Y. (2015). Effects of a DPP4 inhibitor on cisplatin-induced acute kidney injury: Study protocol for a randomized controlled trial. Trials.

[14] El-Ghiaty M.A., Ibrahim O.M.H., Abdou S.M., Hussein F.Z. (2014). Evaluation of the protective effect of Cystone^®^ against cisplatin-induced nephrotoxicity in cancer patients, and its influence on cisplatin antitumor activity. Int. Urol. Nephrol..

[15] Shahbazi F., Sadighi S., Dashti-Khavidaki S., Shahi F., Mirzania M., Abdollahi A., Ghahremani M.-H. (2015). Effect of Silymarin Administration on Cisplatin Nephrotoxicity: Report from A Pilot, Randomized, Double-Blinded, Placebo-Controlled Clinical Trial. Phytother. Res..

[16] Karademir L.D., Dogruel F., Kocyigit I., Yazici C., Unal A., Sipahioglu M.H., Oymak O., Tokgoz B. (2016). The efficacy of theophylline in preventing cisplatin-related nephrotoxicity in patients with cancer. Ren. Fail..

[17] Momeni A., Hajigholami A., Geshnizjani S., Kheiri S. (2015). Effect of Silymarin in the Prevention of Cisplatin Nephrotoxicity, a Clinical Trial Study. J. Clin. Diagn. Res..

[18] Matsui M., Saito Y., Yamaoka S., Yokokawa Y., Morikawa Y., Makimoto A., Yuza Y. (2018). Kidney-protective effect of magnesium supplementation in cisplatin-containing chemotherapy for pediatric cancer: A retrospective study. J. Pediatr. Hematol. Oncol..

[19] Busari A.A., Adejare A.A., Shodipe A.F., Oduniyi O.A., Ismail-Badmus K.B., Oreagba I.A. (2018). Protective but Non-Synergistic Effects of Nigella Sativa and Vitamin E against Cisplatin-Induced Renal Toxicity and Oxidative Stress in Wistar Rats. Drug Res..

[20] Guo Y., Wang M., Mou J., Zhao Z., Yang J., Zhu F., Pei G., Zhu H., Wang Y., Xu G. (2018). Pretreatment of Huaiqihuang extractum protects against cisplatin-induced nephrotoxicity. Sci. Rep..

[21] Lee D., Yu J.S., Lee S.R., Hwang G.S., Kang K.S., Park J.G., Kim H.Y., Kim K.H., Yamabe N. (2018). Beneficial Effects of Bioactive Compounds in Mulberry Fruits against Cisplatin-Induced Nephrotoxicity. Int. J. Mol. Sci..

[22] Kandemir F.M., Yildirim S., Caglayan C., Kucukler S., Eser G. (2019). Protective effects of zingerone on cisplatin-induced nephrotoxicity in female rats. Environ. Sci. Pollut. Res..

[23] Kpemissi M., Eklu-Gadegbeku K., Veerapur V.P., Negru M., Taulescu M., Chandramohan V., Hiriyan J., Banakar S.M., Nv T., Suhas D.S. (2019). Nephroprotective activity of Combretum micranthum G. Don in cisplatin induced nephrotoxicity in rats: In-vitro, in-vivo and in-silico experiments. Biomed. Pharmacother..

[24] Ridzuan N.R.A., Rashid N.A., Othman F., Budin S.B., Hussan F., Teoh S.L. (2019). Protective Role of Natural Products in Cisplatin-Induced Nephrotoxicity. Mini Rev. Med. Chem..

[25] Batiha G.E.-S., Olatunde A., El-Mleeh A., Hetta H.F., Al-Rejaie S., Alghamdi S., Zahoor M., Magdy B.A., Murata T., Zaragoza-Bastida A. (2020). Bioactive Compounds, Pharmacological Actions, and Pharmacokinetics of Wormwood (*Artemisia absinthium*). Antibiotics.

[26] Bora K.S., Sharma A. (2010). Neuroprotective effect of Artemisia absinthium L. on focal ischemia and reperfusion-induced cerebral injury. J. Ethnopharmacol..

[27] Craciunescu O., Constantin D., Gaspar-Pintiliescu A., Toma L., Utoiu E., Moldovan L. (2012). Evaluation of antioxidant and cytoprotective activities of *Arnica montana* L. and *Artemisia absinthium* L. ethanolic extracts. Chem. Central J..

[28] Lee Y.-J., Thiruvengadam M., Chung I.-M., Nagella P. (2013). Polyphenol composition and antioxidant activity from the vegetable plant *Artemisia absinthium* L. Aust. J. Crop. Sci..

[29] Krebs S., Omer B., Omer T.N., Fliser D. (2010). Wormwood (*Artemisia absinthium*) for Poorly Responsive Early-Stage IgA Nephropathy: A Pilot Uncontrolled Trial. Am. J. Kidney Dis..

[30] Omer B., Krebs S., Omer H., Noor T. (2007). Steroid-sparing effect of wormwood (*Artemisia absinthium*) in Crohn’s disease: A double-blind placebo-controlled study. Phytomedicine.

[31] Krebs S., Omer T.N., Omer B. (2010). Wormwood (Artemisia absinthium) suppresses tumour necrosis factor alpha and accelerates healing in patients with Crohn’s disease—A controlled clinical trial. Phytomedicine.

[32] Radwan R.R., Abdel Fattah S.M. (2017). Mechanisms involved in the possible nephroprotective effect of rutin and low dose gamma irradiation against cisplatin-induced nephropathy in rats. J. Photochem. Photobiol. B.

[33] Basu A., Bhattacharjee A., Hajra S., Samanta A., Bhattacharya S. (2016). Ameliorative effect of an oxovanadium (IV) complex against oxidative stress and nephrotoxicity induced by cisplatin. Redox Rep..

[34] Sun C.-Y., Nie J., Zheng Z.-L., Zhao J., Wu L.-M., Zhu Y., Su Z.-Q., Zheng G.-J., Feng B. (2019). Renoprotective effect of scutellarin on cisplatin-induced renal injury in mice: Impact on inflammation, apoptosis, and autophagy. Biomed. Pharmacother..

[35] Farzaneh F., Ebrahim H.S., Akbar V. (2015). Investigating on Effect of Wormwood Extract on Reduction of Renal Toxicity in Treated Rats by Azathioprine. Biomed. Pharmacol. J..

[36] Mukhopadhyay P., Horváth B., Zsengellér Z., Zielonka J., Tanchian G., Holovac E., Kechrid M., Patel V., Stillman I.E., Parikh S.M. (2012). Mitochondrial-targeted antioxidants represent a promising approach for prevention of cisplatin-induced nephropathy. Free Radic. Biol. Med..

[37] Karasawa T., Steyger P.S. (2015). An integrated view of cisplatin-induced nephrotoxicity and ototoxicity. Toxicol. Lett..

[38] Hwang B.S., Lee D., Choi P., Kim K.S., Choi S.-J., Song B.G., Kim T., Song J.H., Kang K.S., Ham J. (2018). Renoprotective Effects of Hypoxylonol C and F Isolated from Hypoxylon truncatum against Cisplatin-Induced Cytotoxicity in LLC-PK1 Cells. Int. J. Mol. Sci..

[39] Jung K., Lee D., Yu J.S., Namgung H., Kang K.S., Kim K.H. (2016). Protective effect and mechanism of action of saponins isolated from the seeds of gac (Momordica cochinchinensis Spreng.) against cisplatin-induced damage in LLC-PK1 kidney cells. Bioorgan. Med. Chem. Lett..

[40] Lee D., Lee S.R., Kang K.S., Kim K.H. (2020). Benzyl salicylate from the stems and stem barks of Cornus walteri as a nephroprotective agent against cisplatin-induced apoptotic cell death in LLC-PK1 cells. RSC Adv..

[41] Volarevic V., Djokovic B., Jankovic M.G., Harrell C.R., Fellabaum C., Djonov V., Arsenijevic N. (2019). Molecular mechanisms of cisplatin-induced nephrotoxicity: A balance on the knife edge between renoprotection and tumor toxicity. J. Biomed. Sci..

[42] Dickey D.T., Muldoon L.L., Doolittle N.D., Peterson D.R., Kraemer D.F., Neuwelt E.A. (2008). Effect of N-acetylcysteine route of administration on chemoprotection against cisplatin-induced toxicity in rat models. Cancer Chemother. Pharmacol..

[43] Sooriyaarachchi M., Narendran A., Gailer J. (2013). N-Acetyl-l-cysteine modulates the metabolism of cis-platin in human plasma in vitro. Metallomics.

[44] Emir S., Özyörük D., Arman Ö., Özbek N., Tunç B. (2016). Accidental cisplatin overdose in a child: Successful management with repetitive plasmapheresis and use of chemoprotective agents. Turk. J. Pediatr..

[45] Daradka H.M., Abas M.M., Mohammad M.A.M., Jaffar M.M. (2014). Antidiabetic effect of *Artemisia absinthium* extracts on alloxan-induced diabetic rats. Comp. Haematol. Int..

[46] Kharoubi O., Slimani M., Aoues A., Seddik L. (2008). Prophylactic effects of wormwood on lipid peroxidation in an animal model of lead intoxication. Indian J. Nephrol..

[47] Ali A., Rahman K., Jahan N., Jamil A., Rashid A., Shah S.M.A. (2016). Protection of DNA during oxidative stress and cytotoxic potential of *Artemisia absinthium*. Pak. J. Pharm. Sci..

[48] Ummat A., Rechkoblit O., Jain R., Choudhury J.R., Johnson R.E., Silverstein T.D., Buku A., Lone S., Prakash L., Prakash S. (2012). Structural basis for cisplatin DNA damage tolerance by human polymerase η during cancer chemotherapy. Nat. Struct. Mol. Biol..

[49] Hu J., Lieb J.D., Sancar A., Adar S. (2016). Cisplatin DNA damage and repair maps of the human genome at single-nucleotide resolution. Proc. Natl. Acad. Sci. USA.

[50] Kamali H., Mohammadi A., Sani T.A., Ameri A.A., Imani M., Golmakani E. (2015). Seasonal variation in the chemical composition, antioxidant activity, and total phenolic content of *Artemisia absinthium* essential oils. Pharmacogn. Res..

[51] Bora K.S., Sharma A. (2011). Evaluation of antioxidant and free-radical scavenging potential of *Artemisia absinthium*. Pharm. Biol..

[52] Shafi G., Hasan T.N., Syed N.A., Al-Hazzani A.A., Alshatwi A.A., Jyothi A., Munshi A. (2012). *Artemisia absinthium* (AA): A novel potential complementary and alternative medicine for breast cancer. Mol. Biol. Rep..

[53] Koyuncu I. (2018). Evaluation of anticancer, antioxidant activity and phenolic compounds of *Artemisia absinthium* L. Extract. Cell. Mol. Biol..

[54] Nazeri M., Mirzaie-Asl A., Saidijam M., Moradi M. (2020). Methanolic extract of *Artemisia absinthium* prompts apoptosis, enhancing expression of Bax/Bcl-2 ratio, cell cycle arrest, caspase-3 activation and mitochondrial membrane potential destruction in human colorectal cancer HCT-116 cells. Mol. Biol. Rep..

[55] Sultan M.H., Zuwaiel A.A., Sivakumar S., Alshahrani S., Alqahtani S.S., Madkhali O., Elmobark M.E. (2020). Bioactive principles and potentiality of hot methanolic extract of the leaves from *Artemisia absinthium* L “in vitro cytotoxicity against human MCF-7 breast cancer cells, antibacterial study and wound healing activity. Curr. Pharm. Biotechnol..

[56] Ali M., Abbasi B.H. (2013). Thidiazuron-Induced Changes in Biomass Parameters, Total Phenolic Content, and Antioxidant Activity in Callus Cultures of *Artemisia absinthium* L. Appl. Biochem. Biotechnol..

[57] Seigler D.S. (2012). Plant Secondary Metabolism.

[58] Saltveit M.E. (2017). Synthesis and Metabolism of Phenolic Compounds. Fruit and Vegetable Phytochemicals: Chemistry and Human Health.

[59] Bochkov D.V., Sysolyatin S.V., Kalashnikov A.I., Surmacheva I.A. (2012). Shikimic acid: Review of its analytical, isolation, and purification techniques from plant and microbial sources. J. Chem. Biol..

[60] Al-Malki A.L. (2019). Shikimic acid from Artemisia absinthium inhibits protein glycation in diabetic rats. Int. J. Biol. Macromol..

[61] Nieskens T.T.G., Peters J.G.P., Dabaghie D., Korte D., Jansen K., Van Asbeck A.H., Tavraz N.N., Friedrich T., Russel F.G.M., Masereeuw R. (2018). Expression of Organic Anion Transporter 1 or 3 in Human Kidney Proximal Tubule Cells Reduces Cisplatin Sensitivity. Drug Metab. Dispos..

[62] Manna K., Khan A., Das D.K., Kesh S.B., Das U., Ghosh S., Dey R.S., Saha K.D., Chakraborty A., Chattopadhyay S. (2014). Protective effect of coconut water concentrate and its active component shikimic acid against hydroperoxide mediated oxidative stress through suppression of NF-κB and activation of Nrf2 pathway. J. Ethnopharmacol..

[63] Güntürk I., Yazici C., Köse S.K., Dağli F., Yücel B., Yay A.H. (2019). The effect of N-acetylcysteine on inflammation and oxidative stress in cisplatin induced nephrotoxicity: A rat model. Turk. J. Med Sci..

[64] Jing T., Liao J., Shen K., Chen X., Xu Z., Tian W., Wang Y., Jin B., Pan H. (2019). Protective effect of urolithin a on cisplatin-induced nephrotoxicity in mice via modulation of inflammation and oxidative stress. Food Chem. Toxicol..

[65] Ma N., Wei W., Fan X., Ci X. (2019). Farrerol Attenuates Cisplatin-Induced Nephrotoxicity by Inhibiting the Reactive Oxygen Species-Mediated Oxidation, Inflammation, and Apoptotic Signaling Pathways. Front. Physiol..

[66] Deniz G.Y., Laloglu E., Altun S., Yiğit N., Gezer A. (2020). Antioxidant and anti-apoptotic effects of vitexilactone on cisplatin-induced nephrotoxicity in rats. Biotech. Histochem..

[67] Sadeghi H., Mansourian M., Panahi kokhdan E., Salehpour Z., Sadati I., Abbaszadeh-Goudarzi K., Asfaram A., Doustimotlagh A.H. (2020). Antioxidant and protective effect of Stachys pilifera Benth against nephrotoxicity induced by cisplatin in rats. J. Food Biochem..

[68] Rabelo T.K., Zeidán-Chuliá F., Caregnato F.F., Schnorr C.E., Gasparotto J., Serafini M.R., Araújo A.A.D.S., Quintans J.D.S.S., Moreira J.C.F., Gelain D.P. (2015). In Vitro Neuroprotective Effect of Shikimic Acid Against Hydrogen Peroxide-Induced Oxidative Stress. J. Mol. Neurosci..

[69] Lieberthal W., Triaca V., Levine J. (1996). Mechanisms of death induced by cisplatin in proximal tubular epithelial cells: Apoptosis vs. necrosis. Am. J. Physiol. Physiol..

[70] Topcu-Tarladacalisir Y., Sapmaz-Metin M., Karaca T. (2016). Curcumin counteracts cisplatin-induced nephrotoxicity by preventing renal tubular cell apoptosis. Ren. Fail..

[71] Lee R.H., Song J.M., Park M.Y., Kang S.K., Kim Y.K., Jung J.S. (2001). Cisplatin-induced apoptosis by translocation of endogenous Bax in mouse collecting duct. Biochem. Pharmacol..

[72] Tang L.-L., Ye J.-Y., Jiang S.-N., Zheng J.-S. (2017). 3,4-oxo-isopropylidene-shikimic acid inhibits cerebral ischemia-induced oxidative stress and neuronal apoptosis in rats. Am. J. Transl. Res..

[73] Chen X., Li X., Zhai X., Zhi X., Cao L., Qin L., Su J. (2018). Shikimic Acid Inhibits Osteoclastogenesis In Vivo and In Vitro by Blocking RANK/TRAF6 Association and Suppressing NF-kappaB and MAPK Signaling Pathways. Cell Physiol. Biochem..

[74] McMahon K.R., Rassekh S.R., Schultz K.R., Blydt-Hansen T., Cuvelier G.D.E., Mammen C., Pinsk M., Carleton B.C., Tsuyuki R.T., Ross C.J.D. (2020). Epidemiologic Characteristics of Acute Kidney Injury During Cisplatin Infusions in Children Treated for Cancer. JAMA Netw. Open.

[75] McMahon K.R., Harel-Sterling M., Pizzi M., Huynh L., Hessey E., Zappitelli M. (2018). Long-term renal follow-up of children treated with cisplatin, carboplatin, or ifosfamide: A pilot study. Pediatr. Nephrol..

[76] Holditch S.J., Brown C.N., Lombardi A.M., Nguyen K.N., Edelstein C.L. (2019). Recent Advances in Models, Mechanisms, Biomarkers, and Interventions in Cisplatin-Induced Acute Kidney Injury. Int. J. Mol. Sci..

[77] Arunkumar P.A., Viswanatha G.L., Radheshyam N., Mukund H., Belliyappa M.S. (2012). Science behind cisplatin-induced nephrotoxicity in humans: A clinical study. Asian Pac. J. Trop. Biomed..

[78] Geyikoglu F., Emir M., Colak S., Koc K., Turkez H., Bakır M., Hosseinigouzdagani M., Cerig S., Keles O.N., Ozek N.S. (2017). Effect of oleuropein against chemotherapy drug-induced histological changes, oxidative stress, and DNA damages in rat kidney injury. J. Food Drug Anal..

[79] Saito Y., Okamoto K., Kobayashi M., Narumi K., Furugen A., Yamada T., Iseki K. (2017). Magnesium co-administration decreases cisplatin-induced nephrotoxicity in the multiple cisplatin administration. Life Sci..

[80] Won A.J., Kim S., Kim Y.G., Kim K.-B., Choi W.S., Kacew S., Kim K.S., Jung J.H., Lee B.M., Kim S. (2016). Discovery of urinary metabolomic biomarkers for early detection of acute kidney injury. Mol. BioSyst..

[81] Tamura Y., Ikeda O., Nakasone Y., Iryo Y., Yamashita Y. (2010). Effect of sodium thiosulfate on cisplatin removal after intra-arterial embolization with a lipiodol-platinum suspension for hepatocellular carcinoma. Acta Radiol..

[82] Farrell N., Roberts J.D., Hacker M.P. (1991). Shikimic acid complexes of platinum. Preparation, reactivity, and antitumor activity of (R,R-1,2-diaminocyclohexane) bis(shikimato)platinum(II). Evidence for a novel rearrangement involving platinum-carbon bond formation. J. Inorg. Biochem..

[83] Lee D., Lee D.S., Jung K., Hwang G.S., Lee H.L., Yamabe N., Lee H.J., Eom D.W., Kim K.H., Kang K.S. (2018). Protective effect of ginsenoside Rb1 against tacrolimus-induced apoptosis in renal proximal tubular LLC-PK1 cells. J. Ginseng Res..

[84] Lim L., Ju S., Song H. (2019). *Dendropanax morbifera* extract protects cardiomyocytes against hypoxia/reoxygenation injury by inhibition of reactive oxygen species generation and calcium perturbation. Nat. Prod. Sci..

[85] Kim D.H., Kim D.W., Jung B.H., Lee J.H., Lee H., Hwang G.S., Kang K.S., Lee J.W. (2019). Ginsenoside Rb2 suppresses the glutamate-mediated oxidative stress and neuronal cell death in HT22 cells. J. Ginseng Res..

[86] Trinh T.A., Park E.-J., Lee D., Song J.H., Lee H.L., Kim K.H., Kim Y., Jung K., Kang K.S., Yoo J.-E. (2019). Estrogenic activity of sanguiin H-6 through activation of estrogen receptor α coactivator-binding site. Nat. Prod. Sci..

[87] R Core Team (2018). R: A Language and Environment for Statistical Computing.

